# Zoonotic Schistosomiasis: The Crossroads Between Animal and Human Schistosomiasis in Africa: Narrative Review

**DOI:** 10.1002/hsr2.71555

**Published:** 2025-11-20

**Authors:** Fatima Amponsah Fordjour, Alexander Kwarteng

**Affiliations:** ^1^ Department of Microbiology University for Development Studies, UDS Tamale Ghana; ^2^ Department of Biochemistry and Biotechnology Kwame Nkrumah University of Science and Technology, KNUST Kumasi Ghana; ^3^ Kumasi Centre for Collaborative Research in Tropical Medicine Kwame Nkrumah University of Science and Technology, KNUST Kumasi Ghana

**Keywords:** mass drug administration, schistosomiasis, zoonosis

## Abstract

**Background:**

The fight against schistosomiasis (urogenital and intestinal) has prolonged more than expected with more areas that were previously known to have less or no incidence of infection now recording alarming incidence rate. Researchers and stakeholders are now concerned about what is probably sustaining the possible infection, and one of the leading hypotheses is the zoonotic forms of the species playing a major role.

**Aim:**

In Africa, livestock and their owners stay in proximity, thereby bringing together both human and livestock schistosomes. *Schistosoma haematobium* (human) and *Schistosoma bovis* (livestock) have the same snail host and can result in hybridization during the sexual phase of the parasite development in the mammalian host. These genetic spillovers lead to hybrid formation of schistosomes which can be problematic for diagnosis, treatment, and control programs.

**Conclusion:**

Although there is less data on the ability of hybrid to cause resistance on praziquantel, experts suggest a possibility. This review calls for researchers, experts, and stakeholders to come together to provide empirical data on this menace especially on the prevailing *Schistosoma* species in livestock on the African continent.

**Abbreviations**
FAO: Food and Agriculture Organization
MDA: mass drug administration
NTDs: neglected tropical diseases
OIE: World Organization for Animal Health
TAS: transmission assessment survey
UNEP: United Nations Environment Program
WHO: World Health Organization

## 1 Introduction

The World Health Organization (WHO) has targeted eliminating almost all neglected tropical diseases (NTDs) through chemotherapy, vector control, and other strategies [1]. However, these elimination programs have mainly targeted human infections, with limited attention on livestock infections that could be transmissible to humans (zoonosis). Recent empirical evidence suggests that in addition to *Schistosoma* species for which humans are the recognized mammalian host, livestock schistosomes are also infective to humans [2, 3]. This means there is a higher possibility of hybrid formation between animal and human schistosomes [4], especially during the sexual reproduction of the parasite in the definitive host. Over the years, little or no emphasis has been placed on controlling schistosomes transmissible from livestock to humans (i.e., zoonotic schistosomiasis). As a result, this has increased the risk of transmission and recrudescence in many regions. This is particularly relevant to high transmission areas where the coexistence of people and livestock overlaps with the distribution of suitable snail intermediate hosts. For many years, *Schistosoma* species have been largely considered to have limited zoonotic potential. Nonetheless, studies have shown the possible zoonotic potential of animal schistosomes and their ability to form hybrids with human schistosomes [5, 6].

**Figure 1:**
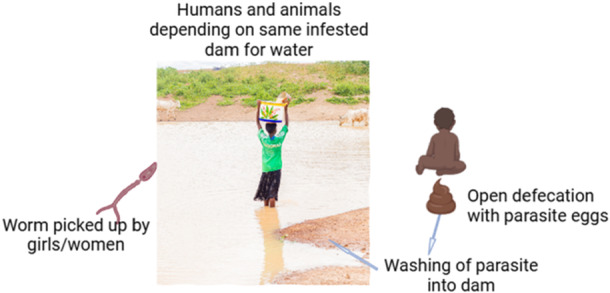
Humans and animals depend on the same water source.

In Africa, and especially in Ghana, livestock and their owners stay close as these animals drink from the same water bodies the communities depend upon (Figure 1). Furthermore, there is an increasing number of reports on the presence of hybrid species, with characteristics between previously assumed human‐specific and animal‐specific schistosomes [4, 7]. Praziquantel is a safe and highly efficacious drug but could become ineffective if resistance emerges. To reach the revised WHO goal of eliminating schistosomiasis by 2030, new strategies should be implemented to counter the role of animal reservoirs in perpetuating transmission.

Schistosomiasis also affects dairy production and the general growth of livestock, and therefore attempt must be made on the possible inclusion of livestock in mass drug administration (MDA) using praziquantel as a measure of food security [2]. Nevertheless, there is limited data on treating livestock with praziquantel, especially in Africa, as existing data on hybrid schistosomes are mainly found in species specific to other continents. However, limited attention has been paid to the role of livestock as reservoir hosts for both hybrid and human forms of the disease, and the prevalence of transmission of schistosomes to humans via farmed animals. This review calls for opinions on the inclusion of livestock in MDA and to set strategies that could mitigate possible zoonotic and hybrid schistosomiasis, especially in Africa.

## 2 Epidemiology of Schistosomiasis

Schistosomiasis is transmitted through freshwater‐infected snails to humans or animals in contact with water [8]. Schistosomiasis is endemic in 78 countries globally, with an estimated 250 million people infected, of whom 120 million are symptomatic, 20 million severely diseased and an estimated number of 200,000 deaths per year [9]. The disease is endemic in Ghana, being widespread across all regions of the country [10]. *Schistosoma haematobium* (*S. haematobium*; urogenital schistosomiasis) is highly endemic in all parts of the country while *Schistosoma mansoni* (*S. mansoni*; intestinal schistosomiasis) is highly endemic in the western and upper east regions of Ghana. Until 2008, the prevalence of schistosomiasis in Ghana had not been fully documented [11], though *S. haematobium* (urogenital schistosomiasis) was recognized to be more prevalent than *S. mansoni* in the country [1, 12].

Schistosomiasis disease is a common parasitic disease among cattle production in Africa with about 165 million cattle affected globally, this notwithstanding, it hardly infects other domestic animals such as sheep and goat; nor does it have any serious effect on wild rodents and primates [13, 14]. The disease is of veterinary and economic significance [14]. Almost 1.5 million cattle suffer from schistosomiasis in China with more than 5 million are at risk of infection [15], however, data on diseased cattle in Africa are limited. Schistosomiasis in livestock usually presents with no clinical manifestation, nonetheless, persisting infection results in enteritis and anemia, reducing productivity (meat and dairy) significantly [5].

**Figure 2:**
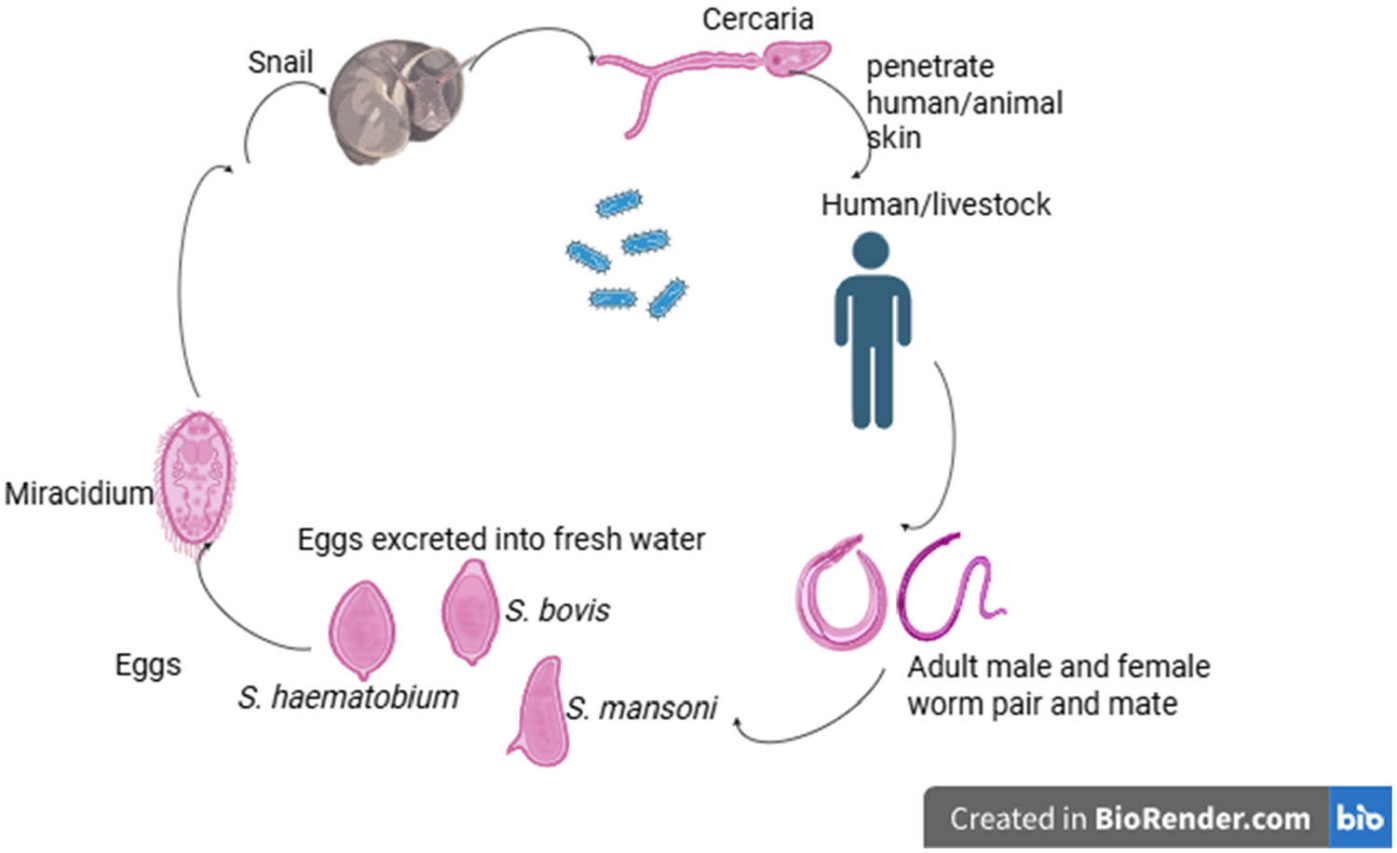
Lifecycle diagram of *Schistosoma* species.

There has been less or no data on the livestock schistosomiasis in Ghana. Several attempts to estimate the human population infected or at risk of contracting schistosomiasis in Ghana have been made in the last three decades. However, in 2008, it was estimated that 8.5 million were at high risk of infection with schistosomiasis out of a total population of about 23.8 million in Ghana, while in 2010, this number increased to 9.5 million [10]. Individuals are infected upon daily contact (bathing, washing, swimming, and farming) with water infested with snails. Most endemic communities have limited or no access to safe water, sanitation, and proper hygiene; hence, there is the possibility of humans and animals depending on the same source of water. Indiscriminate defecation and urination in open water due to lack of proper hygiene conditions allow schistosome eggs to encounter snail hosts. Transmission ensues when humans have sufficient contact with contaminated water bodies containing schistosome infective larvae (cercariae). The transmission pattern of the infection is shown in Figure 2.

## 3 Transmission and Control

Until 2008, the prevalence of schistosomiasis in Ghana had not been fully documented [11], though *S. haematobium* (urogenital schistosomiasis) was recognized to be more prevalent than *S. mansoni* in the country [1, 12].

The transmission of schistosomiasis can be controlled by reducing contact with freshwater infested with parasite larvae. At least five trematode species are known to infect humans. These are *S. haematobium* (*Bulinus* spp. snails), *S. mansoni* (*Biomphalaria* spp. snails), *Schistosoma intercalatum* (*S. intercalatum*), *Schistosoma japonicum* (*S. japonicum*; *Oncomelania* spp. snails), *S. mansoni,* and *Schistosoma mekongi* (*S. mekongi*; *Neutricula* spp. snails) [16]. Among the livestock forms (*Schistosoma curassoni* [*S. curassoni*] and *Schistosoma mattheei* [*S. mattheei*]) of the infection *Schistosoma bovis* (*S. bovis*) is the commonest reported usually in cattle [2].

Vertebrates as reservoirs for *S. japonicum* (mostly mammals and rodents) have been found to aid in sustaining transmission of this zoonotic parasite [17, 18]. There is evidence supporting the introduction of *S. mansoni* from West Africa via the transatlantic slave trade into other continents [19], in these regions, transmission can be sustained by rodents reservoirs even in the absence of human host.

Undoubtedly, the disease is mostly prevalent in areas without segregated water systems for both humans and animals and adequate sanitation.

Currently, the WHO prevention guidelines for schistosomiasis include school‐going children with praziquantel (PZQ), creating awareness on the transmission of the infection, and ultimately calling on governments and nongovernmental organizations to help in the provision of segregated water systems for these communities. Vector control has been one of the approaches targeted at prevention of this infection, however, the onset of resistance of some snail species to molluscicides calls for a better understanding of the process of disease transmission and a development of integrated control strategies for the prevention and control of schistosomiasis [20].

## 4 Zoonosis and Hybrid Schistosomes

The importance of zoonotic and hybrid schistosomiasis transmission can be estimated based on the threshold of individuals affected and are in need of treatment worldwide. *Schistosoma* species have two hosts, usually an asexual phase (intermediate) in a freshwater snail and a sexual phase (definitive host) in mammalian host. Schistosomes are mostly host‐specific found within specific locations to maintain barriers between species. Nonetheless, travel, settlement, increased livestock trade, human activities, and migration facilitate heterogenous crosses between different species thereby giving opportunity to species hybridization during the sexual phase in the mammalian host. The above facilitates hybridization and zoonotic effect on human populations.

Advancement in molecular work has given way to greater exploration of interspecies interaction, specifically between *S. haematobium* responsible for human urogenital schistosomiasis and its sister species *S. bovis* in livestock [21]. Other forms of livestock species such as *S. curassoni* and *S*. *mattheei* (causative agents of intestinal schistosomiasis in livestock) can also hybridize with *S. haematobium*.

Phylogenetic analysis has shown that *S. haematobium* and *S. bovis* are closely related by having freshwater snails of the genus *Bulinus* as intermediate host for both species [22, 23]. Cohost of *S. haematobium* and *S. bovis* by *Bulinus* snails can result in hybridization during the sexual phase of the species in their mammalian host, influencing disease transmission and sustenance. Several studies have already been conducted on *S. haematobium* × *S. bovis* hybrids, with identified samples collected from school‐aged children in Niger [24], Senegal [25, 26, 27], Mali [28], Côte d'Ivoire [29], and some other countries.

Hybridization can complicate prevention programs, diagnosis, and other aspects of the disease; the hybrid forms may show high virulence/resistance compared to real forms of the species. Detail in the epidemiological and pathological manifestation of hybridized schistosomes is still unknown and therefore call for further investigations into the potential zoonotic ability and its impact on treatment programs. Hence, there is the need for an approach that can collectively handle all the sectors from which schistosomiasis can cause an infection and public health concern.

## 5 Praziquantel Resistance

Praziquantel is the WHO‐approved drug of choice for MDA against schistosomiasis in endemic countries. Research has shown praziquantel to be relatively safe, well‐absorbed, and effective oral drug against schistosome species [30]. The mechanism of action of the drug usually starts 1 h after intake by immobilizing the worm and destroying the tegument [30]. Unfortunately, praziquantel has almost no effect on the eggs and larvae, hence, the drug is taken annually or biannually in endemic countries as there is reinfection after treatment. There is therefore fear of resistance in individuals in endemic countries as the drug must be administered repeatedly over several years to be able to finally interrupt transmission in a community [7].

Praziquantel taken through the MDA program is to interrupt the transmission cycle of the infection in endemic communities over time. This means endemic countries must continue to take MDA for several years before they can be certified for elimination. Globally (Brazil, Cambodia, China, Egypt, Mauritius, Iran, Oman, Jordan, Saudi Arabia, Morocco, Tunisia, Nigeria, Ghana, and others), schistosomiasis control has been successfully implemented in so many countries for more than four decades now. Many countries have been able to implement scale‐up programs against schistosomiasis as others are still struggling with the disease [31].

In the sub‐Saharan region, there have been scale‐up interventions programs for over a decade now, together with the introduction of transmission assessment surveys (TASs) to certify the elimination of the disease in countries that have successfully implemented the program. These treatment campaigns have decreased the prevalence of schistosomiasis in school‐age children by almost 60% [30] since its inception.

Praziquantel is efficacious against *S. mansoni, S. haematobium*, and *S. intercalatum* using praziquantel 40 mg/kg/day [30, 32]. Treatment usually removes the parasite, however, there is the possibility of reinfection if individuals continue to stay in endemic communities over time. The response of the drug may vary depending on parasite load. *S. japonicum* and *S. mekongi* which are known to be endemic on the Asian continent require a higher dose of 60 mg/kg/day [31, 33]. Praziquantel has proven to have no risk in pregnancy in animal studies, nonetheless, limited data are available in human studies, and as a result, WHO has declared praziquantel to be a pregnancy Category B drug. The WHO, therefore, supports its use in pregnancy [34, 35, 36]. Mothers who are breastfeeding are also allowed to take praziquantel during MDA program. It is important to note that the benefit versus risk of the drug has not been assessed in children under 4 years and hence is not allowed in toddlers.

The dependence on a single drug in treating schistosomiasis, which a disease of this magnitude is overly disturbing should develop drug resistance. Therefore, there is a need to detect and track resistant *Schistosoma* spp. to face the threat of drug resistance which could impede the WHO 2030 NTD roadmap targets. Thus, there is an urgent need to detect and track resistant *Schistosoma* parasites to investigate the threat of drug resistance to schistosomiasis control programs [37, 38, 39].

## 6 Livestock's Inclusion in the Mass Drug Administration (MDA)

The failure of the elimination goal by 2025 has led to many hypotheses, with the role of livestock schistosomes gaining attention in prolonging MDA programmes. To reach the revised WHO goal of eliminating schistosomiasis by 2030 [31], new strategies should be implemented to counter the role of animal reservoirs in perpetuating transmission in humans. Areas with a lack of segregated water systems for both livestock and humans usually suffer this occurrence as the community and livestock all drink from the same source. Another phenomenon is the settlement type, whereby most farmers stay near their livestock. This settlement type is common in sub‐Saharan Africa connecting the human and livestock schistosomes.

At the just‐ended 2024 World Food Forum organized by the Food and Agricultural Organization of the United Nations, one key agendum was food security [40]. The impact of schistosomiasis on dairy production and the general growth of livestock is underestimated. This has become a global concern as there is the need to provide good food for all today and tomorrow [40]. The infection has been reported to deteriorate dairy and meat production, and thus, the need to include livestock in MDA using praziquantel if proven successful through field trials. Nevertheless, there is limited data on treating livestock with praziquantel, especially in Africa. Thorough scientific data are needed to validate the successful integration of livestock into MDA using praziquantel. To achieve this, experts and researchers must come together with a good collaboration system to actualize this goal. All these steps can be taken to mitigate the hunger on the African continent to achieve Sustainable Development Goal 2 (SDG 2).

## 7 Outlook on Hybrid Schistosomes

Hybrid schistosome development has recently been hypothesized, however, less field and laboratory data can be found. Transmission of livestock schistosomes to humans was among the least expected phenomena, not to mention the crossing of human and animal schistosomes. Detrimentally, the situation results from livestock having more contact with humans, living together or towners visiting farms and spending most of their time on the farms. Other sources are as a result of increased human population growth, anthropogenic environmental changes, and global movements of humans and animals, potentially increasing crossing among *Schistosoma* species across Africa and the world. Since these events involve species that infect both humans and animals (domestic and wild), researchers have raised concerns about the emergence of potential zoonotic schistosomiasis [41].

Researchers have suggested that the formation of hybrid could pose resistance to the effectiveness of praziquantel in MDA. Probably efficacy of praziquantel against the hybrid schistosome needs further validation through robust field trials to ensure the ability of the drug against the hybrid. This may further lengthen the schistosomiasis elimination program if this hypothesis is poorly evaluated and not reported on time.

## 8 Using the One Health Approach in Schistosomiasis Disease Elimination

NTDs continue to cause hardship and harm to about a billion people worldwide [9], burdening individuals, families, and communities who are already marginalized and disadvantaged [42]. Considering the One Health approach that recognizes the relationship between human, animal, and the environment, this would be the game changer in addressing NTDs sustainably [7]. One Health is a connected unifying approach mainly to balance and optimize the health of people, animals, and ecosystems. Most NTDs, especially schistosomiasis, have a complex lifecycle that usually involves an intermediate host and the environment which usually supports other NTDs. Thus, challenges arising from all these interphases (human, animal, and environment) can be solved using this integrated approach called One Health, especially in treatment strategies.

In zoonotic schistosomiasis, there is the possibility of hybrid development [3, 18]; using the One Health approach, all forms of the parasite could be considered (both human and animal forms) in treatment programs. To operationalize the use of the One Health approach, the Food and Agriculture Organization of the United Nations (FAO), the World Organization for Animal Health (OIE), the United Nations Environment Program (UNEP), and the WHO have considered the importance of One Health, with each unit charging its members to develop strategies that will help in the integration strategy.

In summary, incorporating One Health principles into NTDs elimination is essential for building resilient treatment programs that can withstand emergencies such as pandemics and other outbreaks. This will help communities to respond and recover from disease outbreaks and disasters thereby minimizing their occurrence. In schistosomiasis infection, this approach will be a key factor in interrupting community transmission. By emphasizing interdisciplinary collaboration, comprehensive risk assessment, capacity building, and community engagement will be feasible.

## 9 The WHO Outlooks and constraint on Schistosomiasis Elimination Program

Despite numerous studies that have been carried out on the development of a vaccine against schistosomiasis, there is no licensed vaccine yet, the available option is still using praziquantel to treat infected cases in all endemic countries [43]. Besides praziquantel for MDA, possible strategies for tackling the schistosomiasis, as mentioned above, must be strengthened (snail control, sanitation, and health education). Currently, the goal of elimination of schistosomiasis as a global public health concern has been estimated at 2030. All stakeholders are needed on board to actualize set target [44].

The WHO strategy for schistosomiasis control is for reducing infection through timely and targeted treatment using the community‐based treatment approach with praziquantel (preventive chemotherapy) [37]. Baseline treatment usually requires regular treatment from all at‐risk groups. In countries where the baseline prevalence is not alarming, there are usually targeted groups for treatment with a special focus on preschool‐aged children, school‐aged children, adults considered to be at risk in endemic areas (pregnant women), and people with occupations involving contact with infested water, such as fishermen, farmers, irrigation workers and women whose domestic tasks bring them in regular contact with infested water [45, 46].

The number of treatments annually depends on infection prevalence in school‐going children. In areas with a high incidence of schistosomiasis, treatment may have to be repeated every year for several years.

The fight against all NTDs, especially schistosomiasis, does not end with treatment, it is important to conduct regularized monitoring and evaluation on the occurrence of infection so to assess the sharpness of the control interventions. TASs as prescribed by WHO must be conducted to certify elimination of the disease in endemic areas. Preliminary reports from the field have shown that there is resurgence of the infection in areas that stopped MDA without proper TASs [47].

The main goal of schistosomiasis control program is to reduce transmission and disease morbidity toward its elimination. Timely treatment of individuals in endemic areas only cures mild symptoms and prevents infected people from developing severe, late‐stage chronic diseases such as kidney and liver dysfunction. Hence, the need to provide drugs continually for several years to break the transmission cycle.

A major setback to schistosomiasis control program is the inadequacy of praziquantel, particularly for treating adults. Report shows that globally only 29.9% of affected individuals requiring treatment received treatment in 2021 (43.3% of them been school‐aged children). The above shows a sharp decline of about 38% in the treatment program as compared to 2019. This may be attributed to COVID‐19 pandemic which accounted for the suspension of treatment campaigns of many diseases including schistosomiasis [9].

**Figure 3:**
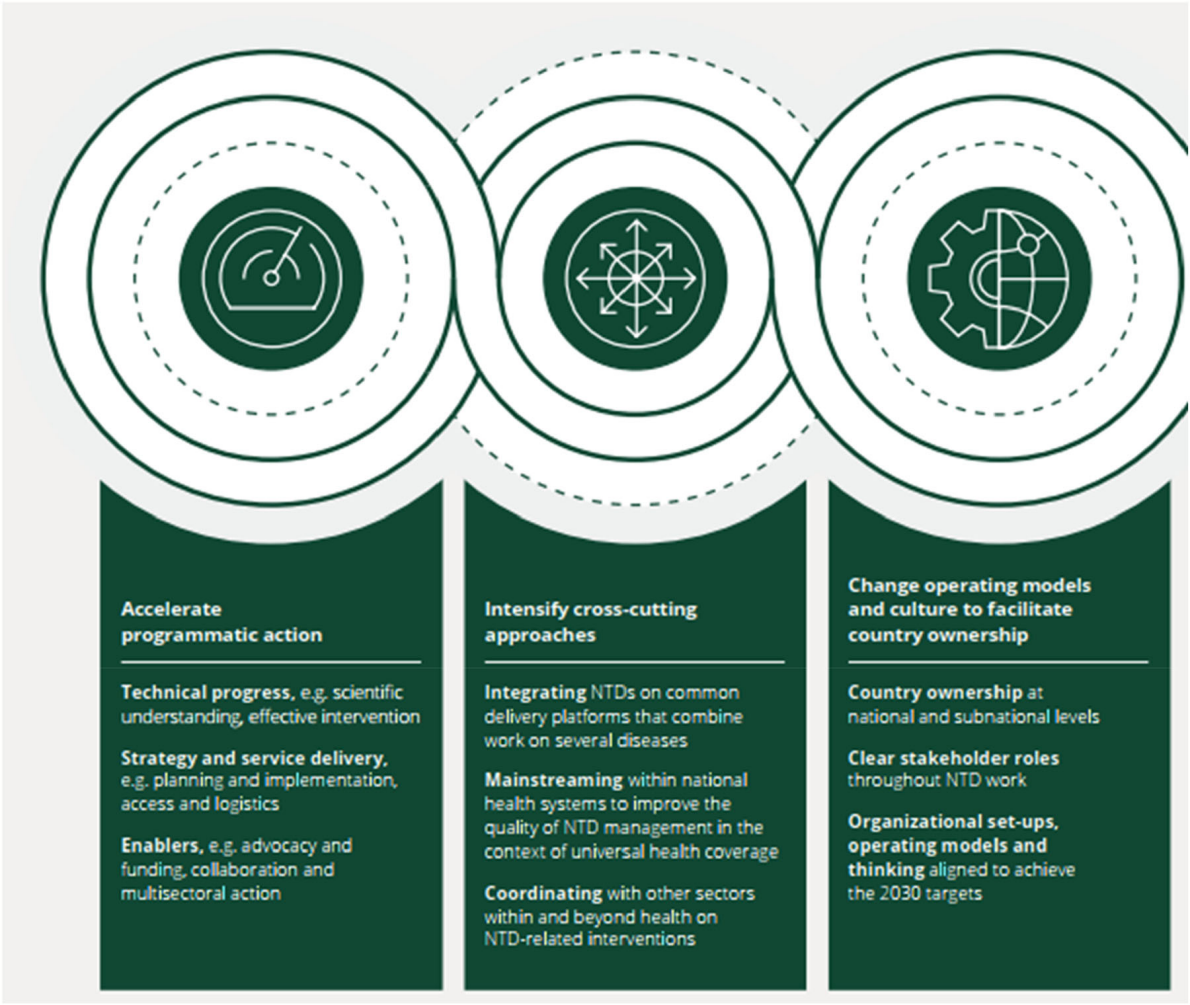
WHO's aims to eliminate the disease as a public health issue by 2030 [9].

In the quest to solve this menace by the WHO, an integrated approach is needed to control NTDs. Although they may all vary medically, NTDs share features that possibly allow them to perpetuate in conditions of poverty. In this quest, the WHO consults and collaborates with other agencies (partners from academic and research institutions, private sector, nongovernmental organizations, international development agencies, and other United Nations organizations) to put up strategies that can serve as technical guidelines and tools for use by national control programs (Figure 3). In the space of collaboration of WHO with other partners and the private sector, the WHO has advocated for increased access to praziquantel by endemic countries together with implementation resources.

## 10 Conclusion

The goal to eliminate schistosomiasis has been ongoing for decades (more than four decades now); however, assessments from field surveys still show considerable prevalence/incidence of infection persisting in endemic communities. This delay further hinders the goal of reaching the SDGs for NTDs. Transmission among human populations with zoonotic and hybrid forms of the schistosomes has been considered an impediment to the success of the roadmap of elimination by 2030. The neglect of animals, especially domestic ones and livestock in schistosomiasis treatment, has also been shown to contribute to the delay in achieving target. There is therefore the need for an integrated approach that can unify the constraints of all possible sources of schistosomiasis transmission so to have a unified approach. One Health as an integrated approach will help in reaching the goal of elimination. This approach will eventually target four SDGs (1, 2, 3, and 6). This approach supports resource‐limited areas where funding for each goal is unavailable, as it interlaces the human, animal, and environment. To reach the set goal of schistosomiasis elimination by 2030, endemic countries must revise their national NTDs structures and incorporate the One Health approach to hasten the process.

## Author Contributions

**Fatima Amponsah Fordjour:** conceptualization, writing – original draft, visualization, writing – review and editing, methodology, data curation. **Alexander Kwarteng:** writing – review and editing, visualization, validation, supervision.

## Ethics Statement

The authors have nothing to report.

## Consent

Consent for publication is not applicable. However, all published articles referred to are appropriately cited.

## Conflicts of Interest

The authors declare no conflicts of interest.

## Transparency Statement

The lead author Fatima Amponsah Fordjour affirms that this manuscript is an honest, accurate, and transparent account of the study being reported; that no important aspects of the study have been omitted; and that any discrepancies from the study as planned (and, if relevant, registered) have been explained.

## Data Availability

The data are accessible via referenced articles. The authors confirm that the data supporting the findings of this study are available within the article.
